# Effect of Semaglutide versus placebo on psychotic symptoms and quality of life - a pre-specified secondary analysis of HISTORI: A randomized clinical trial in people with pre-diabetes and schizophrenia

**DOI:** 10.1192/j.eurpsy.2022.2064

**Published:** 2022-09-01

**Authors:** N. Uhrenholt, A. Ganeshalingam, S. Arnfred, J. Frystyk, P. Gæde, N. Bilenberg

**Affiliations:** 1 Psychiatry West, Region Zealand, Research Unit For Psychotherapy And Psychopathology, Slagelse, Denmark; 2 Odense University Hospital., Department Of Endocrinology M / Steno Diabetes Centre Odense (sdco), Odense, Denmark; 3 Næstved, Slagelse og Ringsted Hospital, Department Of Cardiology And Endocrinology, Slagelse, Denmark; 4 Mental Health Services in the Region of Southern Denmark, Child And Adolescent Psychiatry Odense, Odense C, Denmark

**Keywords:** schizophrénia, Prediabetes, Glucagon-Like Peptide 1, Positive and negative symptoms (PANSS)

## Abstract

**Introduction:**

Life expectancy of people with schizophrenia is reduced by 10-20 years compared to the general population. The excess mortality is part due to an increased prevalence of cardiovascular disease, prediabetes and obesity, which are in part due to antipsychotic treatment. Gaining weight is associated with reduced quality of life and also among the most frequently reported reasons for the discontinuation of treatment. Lifestyle changes have a time limited effect, and therefore, interest has focused on Glucagon-Like Peptide 1 receptor agonist treatment. Semaglutide, currently used to treat type 2-diabetes in doses up to 1.0 mg once-weekly, has shown promising results regarding weight loss in doses up to 2.4 mg once-weekly. It may also be able to reduce the risk of developing diabetes and cardiovascular disease.

**Objectives:**

The HISTORI Trial aims to reduce risk of developing diabetes and cardiovascular disease in people with schizophrenia, prediabetes and overweight and to investigate for an indirect effect of Semaglutide on psychotic symptoms and quality of life through a weight loss.

**Methods:**

A 30 weeks randomized, placebo-controlled, double-blinded study with once-weekly injections of Semaglutide 1.0 mg. Primary inclusion criteria are age 18-40 years, schizophrenia, prediabetes, overweight and treatment with antipsychotics. Questionnaires and interviews regarding psychotic symptoms, quality of life, medication adherence and physical activity will be applied either monthly or every third month.

**Results:**

will not be ready for the congress. A poster outlining the feasibility challenges will be presented.

**Conclusions:**

Perspective: Through weight loss, Semaglutide may indirectly be able to improve quality of life, medication adherence and psychotic symptoms.

**Disclosure:**

No significant relationships.

